# Development of thermostable sucrose phosphorylase by semi-rational design for efficient biosynthesis of alpha-D-glucosylglycerol

**DOI:** 10.1007/s00253-021-11551-0

**Published:** 2021-09-20

**Authors:** Yuanyuan Xia, Xiaoyu Li, Linli Yang, Xiaozhou Luo, Wei Shen, Yu Cao, Lukasz Peplowski, Xianzhong Chen

**Affiliations:** 1 Key Laboratory of Industrial Biotechnology, Ministry of Education, School of Biotechnology, Jiangnan University, 1800 Lihu Avenue, Wuxi, 214122 Jiangsu China; 2 School of Biotechnology, Jiangnan University, Wuxi, 214122 Jiangsu China; 3 Center for Synthetic Biochemistry, Shenzhen Institutes of Advanced Technology, Chinese Academy of Sciences, Shenzhen, 518055 China; 4 Institute of Physics, Faculty of Physics, Astronomy and Informatics, Nicolaus Copernicus University in Torun, Grudziadzka 5, 87-100 Torun, Poland

**Keywords:** Sucrose phosphorylase, Thermostability, Semi-rational design, α-D-glucosylglycerol, Molecular dynamics simulations

## Abstract

**Abstract:**

Sucrose phosphorylase (SPase) can specifically catalyze transglycosylation reactions and can be used to enzymatically synthesize α-D-glycosides. However, the low thermostability of SPase has been a bottleneck for its industrial application. In this study, a SPase gene from *Leuconostoc mesenteroides* ATCC 12,291 (*LmSPase*) was synthesized with optimized codons and overexpressed successfully in *Escherichia coli*. A semi-rational design strategy that combined the FireProt (a web server designing thermostable proteins), structure–function analysis, and molecular dynamic simulations was used to improve the thermostability of *Lm*SPase. Finally, one single-point mutation T219L and a combination mutation I31F/T219L/T263L/S360A (Mut4) with improved thermostability were obtained. The half-lives at 50 °C of T219L and Mut4 both increased approximately two-fold compared to that of wild-type *Lm*SPase (WT). Furthermore, the two variants T219L and Mut4 were used to produce α-D-glucosylglycerol (αGG) from sucrose and glycerol by incubating with 40 U/mL crude extracts at 37 °C for 60 h and achieved the product concentration of 193.2 ± 12.9 g/L and 195.8 ± 13.1 g/L, respectively, which were approximately 1.3-fold higher than that of WT (150.4 ± 10.0 g/L). This study provides an effective strategy for improving the thermostability of an industrial enzyme.

**Key points:**

• *Predicted potential hotspot residues directing the thermostability of LmSPase by semi-rational design*

• *Screened two positive variants with higher thermostability and higher activity*

• *Synthesized α-D-glucosylglycerol to a high level by two screened positive variants*

**Supplementary Information:**

The online version contains supplementary material available at 10.1007/s00253-021-11551-0.

## Introduction

Sucrose phosphorylase (SPase, EC 2.4.1.7) can catalyze the phosphorolysis of sucrose into α-D-glucose 1-phosphate (α-D-G1P) and D-fructose, and it can glycosylate a broad range of acceptors other than phosphate (Goedl et al. 2010). SPase has outstanding potential for biocatalytic conversion of ordinary table sugar into products with attractive properties (Franceus and Desmet 2020), and it is widely used in the fields of food, medicine, and cosmetics (O’Neill and Field 2015). For instance, SPase can be used to synthesize fine chemicals such as α-D-glucosylglycerol (αGG), which is synthesized from one molecule of sucrose and one glycerol. The αGG has a beneficial function as a humectant in cosmetics and has potential as a healthy food material and therapeutic agent (Bolivar et al. 2017).

Industrial carbohydrate conversion is preferably performed at a higher temperature to avoid microbial contamination (Haki and Rakshit 2003). During the production process, SPase with a high thermostability would reduce the production time under high temperatures and remain active after prolonged continuous catalysis. SPase from *L. mesenteroides* ATCC 12,291 (*Lm*SPase) was reported to have high activity to synthesize α-arbutin but poor thermostability, restricting its industrial applications (Yao et al. 2020). In the process of synthesizing αGG, due to the long reaction time, it is usually necessary to keep the temperature at 30 °C (Bolivar et al. 2017; Goedl et al. 2008). Therefore, improving the thermostability of enzymes to enhance their usability in the industry has attracted increasing interest, and thus, various auxiliary methods have been developed to help predict potential thermostable mutants (Sun et al. 2019; Xu et al. 2020). The semi-rational design combines the benefits of directed evolution and rational design and is suitable for modifying proteins without high-throughput determination methods and structure–function understanding (Chica et al. 2005). Many successful examples have reported that changing a few amino acids can significantly improve the protein thermostability by semi-rational design strategies (Cheng et al. 2020; Roth et al. 2017).

In this study, a semi-rational design strategy that combined the FireProt, a web server designed to predict thermostable mutants concerning structural and evolutionary information automatedly, structure–function analysis, and molecular dynamics (MD) simulations were used to improve the thermostability of *Lm*SPase. Several residues which potentially affect the thermostability of *Lm*SPase were selected, and site-directed mutagenesis was performed on the corresponding residues. To test whether the selected mutants can have advantages in industrial applications, we carried out the catalytic synthesis of αGG at a relatively high temperature.

## Materials and methods

### Plasmid construction

The SPase gene from *L. mesenteroides* ATCC 12,291 (*LmSPase*) synthesized with the optimized codon (BankIt2453263 Seq2453263 MZ005078) was inserted between the *Nco*I and *Xho*I restriction enzyme sites of the pET28a plasmid. The recombinant plasmid pET28a-*LmSPase* was expressed in *Escherichia* *coli* BL21(DE3). The site-directed mutation was constructed using the QuikChange method with pET28a-*LmSPase* as the template, and the primers are listed in Table S1. Then, the PCR products were purified and digested by endonuclease *Dpn*I to remove the template plasmid containing the methylation modification. The PCR product was then transformed into competent *E. coli* BL21(DE3) cells with the chemical transformation method. The transformants grown on the plate were picked for sequencing, and the recombinant bacteria with the correct sequence were selected. The recombinant *E. coli* was first cultivated in liquid Luria–Bertani (LB) broth at 37 °C overnight. Then, 0.5 mL of the cultured bacteria were transferred to 50 mL of terminal broth (TB) medium containing 50 μg/mL kanamycin and grown at 37 °C with shaking at 200 rpm. When the culture reached an optical density at 600 nm (OD600) of 0.6, 0.5 mM isopropyl β-D-1-thiogalactoside (IPTG) was added to induce *Lm*SPase expression and cultured at 25 °C for 24 h. The bacteria were collected by centrifugation and ultrasonically lysed to obtain a soluble supernatant. The protein concentration was adjusted to be consistent between samples, and the protein was verified by SDS-PAGE.

### Homology modeling and mutant library construction

For the hotspot residues prediction, the three-dimensional (3D) structure of *Lm*SPase is used as input for FireProt (https://loschmidt.chemi.muni.cz/fireprot/) was obtained through the online prediction website called Iterative Threading ASSEmbly Refinement (I-TASSER, https://zhanglab.ccmb.med.umich.edu/I-TASSER/) (Roy et al. 2010; Yang et al. 2015; Zhang 2008). Then, a new, high-quality homology model was obtained by SWISS-MODEL (Guex et al. 2009; Waterhouse et al. 2018) as a new crystal structure with higher sequence similarity was released after we finished identifying predicted mutants. The *Lm*SPase model obtained by I-TASSER was evaluated with the structure scoring programs SAVES and PROCHECK. The model with higher scores was used as the input file for further analyses. The mutant library was constructed with an in-silico design program FireProt based on the predicted change in Gibbs free energy (ΔΔG) upon mutation. FireProt employed the dataset derived from the ProTherm database to identify the stable single-point mutations either by energy- or evolution-based approaches. After submitting the model PDB file to the FireProt online program, two mutation lists were generated after the evolution- and energy-based calculation approaches, and a third list combined the above two methods. We selected candidate mutants from the first two lists based on the ΔΔG values.

### Purification of LmSPase and its variants

All protein purification steps were performed at 0–4 °C. The harvested cell pellet containing His-tagged enzyme was resuspended in a binding buffer (20 mM sodium phosphate, 0.5 M NaCl, and 5 mM imidazole (pH 7.4)) and lysed by ultrasonication. The supernatants were obtained by centrifugation at 13,000 × *g* for 45 min. The supernatants were filtered through a 0.22-μm pore-size filter. The His-tagged protein was loaded onto a His-Trap affinity chromatography using AKTA Avant (GE Healthcare UK Ltd) and eluted by elution buffer (binding buffer with 0.5 M imidazole). Fractions containing target proteins were analyzed by SDS-PAGE. The purified wild-type *Lm*SPase was loaded onto a Superdex™ 200 10/300 GL column (GE Healthcare, Mickleton, NJ), and the molecular weight was determined and calculated from the standard curve of marker proteins (Oriental Yeast Co., Ltd.).

### Activity assays of LmSPase and its variants

*Lm*SPase catalyzes the reversible phosphorolysis of sucrose to produce α-D-G1P and D-fructose, and the enzyme activity can be measured by determining the fructose produced. *Lm*SPase was assayed in a 1-mL-reaction mixture containing 500 μL of 5% sucrose, 450 μL of 50 mM sodium phosphate buffer (pH 6.5), and 50 μL of *Lm*SPase crude extract or pure enzyme solution (0.01 mg/mL). The mixture was incubated at 30 °C for 10 min and terminated by adding 1.5 mL of 3,5-dinitrosalicylic acid (DNS) and heating in boiling water for 15 min and detected at 540 nm using UV–Vis spectrophotometer. The fructose concentration in the reaction mixture was determined by a fructose standard curve using the same DNS method. The amount of enzyme required to hydrolyze sucrose to release 1 µmol of fructose per minute is defined as one activity unit (U).

### Thermostability assays of LmSPase and its variants

The thermostability of crude *Lm*SPase and its variants was assayed by incubating 0.01 mg/mL crude enzyme extract at 50 °C for 10 min, and the residual activity was tested. The activity of *Lm*SPase without heat treatment was taken as 100%.

The half-lives (*t*_1/2_) of *Lm*SPase and variants were evaluated by incubating 0.01 mg/mL purified enzymes for 90 min in a water bath at 50 °C and testing their residual activity every 10 min. The half-life of each enzyme was calculated by fitting the data points with exponential decay curves.

The melting temperature (*T*_m_) of a protein can be used to describe its thermodynamic stability, and *T*_m_ was determined by nano-differential scanning calorimetry (nano-DSC, TA Instruments, New Castle, DE), which can capture the heat capacity change in the protein during heating. The concentration of *Lm*SPase and its variants was adjusted to 0.5 mg/mL, and the instrument scanned from 20 to 100 °C by the scan rate at 1 °C/min. The heat capacity curve of the sample was analyzed with nano-DSC analysis software by fitting a curve after subtracting the blank control.

### CD measurements of LmSPase and its variants

Circular dichroism (CD) spectroscopy is an acknowledged technique for analyzing the secondary structure of soluble proteins. The CD spectrum of *Lm*SPase and its variants was measured on the MOS-450/AF-CD-STP-A instrument (Bio-Logic, Grenoble, France) from 195 to 250 nm. Each purified enzyme solution was diluted in 50 mM sodium phosphate buffer (pH 6.5) at a protein concentration of 0.2 mg/mL.

### Synthesis of αGG by LmSPase

Recombinant *Lm*SPase was used to transfer a glucose group of sucrose to the acceptor glycerol to produce αGG. The αGG reaction mixture containing 1.2 mol/L sucrose, 2 mol/L glycerol, and 40 U/mL crude extract of *Lm*SPase was incubated at 37 °C in 2-(N-morpholino) ethanesulfonic acid (MES) buffer at pH 6.5. The αGG was analyzed by high-performance liquid chromatography (HPLC) with a refractive index detector (RID) using a Waters XBridge Amide (4.6 mm × 250 mm, 5 μm) column. The mobile phase was composed of 85% acetonitrile and 15% 1‰ ammonia. The concentration of αGG was calculated from the standard curve by extrapolation.

### MD simulations

As an input structure for the MD analysis, the homology model obtained by SWISS-MODEL (Guex et al. 2009; Waterhouse et al. 2018) based on the 6S9V (Franceus et al. 2019) PDB template was used. Amino acid protonation states were determined using the PROPKA3 tool (Olsson et al. 2011) encoded on the PDB2PQR server (Dolinsky et al. 2007, 2004). Mutations were generated using the mutate option in the psfgen tool—part of the NAMD code (Phillips et al. 2005). The water box was generated with 0.15 mol/L Na^+^ and Clˉ. The final simulation system was 91 × 75 × 77 Å.

In each system, the water box was equilibrated during a 1-ns simulation at 300 K, and then 1000 minimization steps for the whole system were performed. Next, for each case, a 100-ns simulation at 300 K with periodic boundary conditions, long-range electrostatics calculated with Ewald summation, and atmospheric pressure calculated using the Langevin algorithm were obtained. The time step was set to 1 fs. As input homology structure models were used, the first 50 ns of the simulation were treated as an equilibration stage. All details for these simulations at 335 K were the same as those for simulations at 300 K, except for the temperature. To heat the temperature from 300 to 335 K, stepwise heating for 45 ps of the simulation was performed.

All MD simulations were calculated using NAMD 2.12 code (Phillips et al. 2005, 2020) with the Charmm27 forcefield (Mackerell et al. 2004; MacKerell et al. 1998). In addition, analysis of 50–100 ns parts of simulations at 300 K and 335 K was performed using VMD (Humphrey et al. 1996) code and homemade scripts.

## Results

### Production of recombinant His-tagged LmSPase

The SPase gene of *L. mesenteroides* ATCC 12,291 was cloned into the pET28a vector after codon optimization and successfully overexpressed in *E. coli* BL21(DE3) (Figure S1A). The *Lm*SPase protein carries a His-tag at the N-terminus with a theoretical molecular mass of 56.8 kDa, and the apparent molecular weight of *Lm*SPase is 63.4 kDa, as determined by size-exclusion chromatography (Figure S1B), which confirmed that *Lm*SPase exists as a monomer, and the purified SPase proteins were used to test their characteristics.

### Homology modeling of LmSPase

The sucrose phosphorylase (PDB ID: 2GDV) was selected as a template because it shared the highest amino acid sequence identity with *Lm*SPase at the beginning of the research. Then, the model served as an input structure for the FireProt server to generate a mutation pool (Musil et al. 2017). Subsequently, to explore the reasons for increased thermostability, a new model was built for MD simulations because a new crystal structure with higher sequence similarity was released in August 2019, which was after we obtained the positive mutants. The crystal structure of sucrose 6F-phosphate phosphorylase from *Thermoanaerobacter thermosaccharolyticum* (Franceus et al. 2019) (PDB ID: 6S9V) has the highest amino acid identity (40%). The homological models were validated by the VERIFY3D server (Eisenberg et al. 1997; Luthy et al. 1992). The SWISS-MODEL and I-TASSER homology models had 99.17% and 82.86% of the amino acids, respectively, with an average 3D-1D score larger than 0.2, and the structure should have at least 80% of the amino acids with an average 3D-1D score larger than 0.2 to pass verification (Eisenberg et al. 1997; Luthy et al. 1992).

### Hotspot residues prediction

**Fig. 1:**
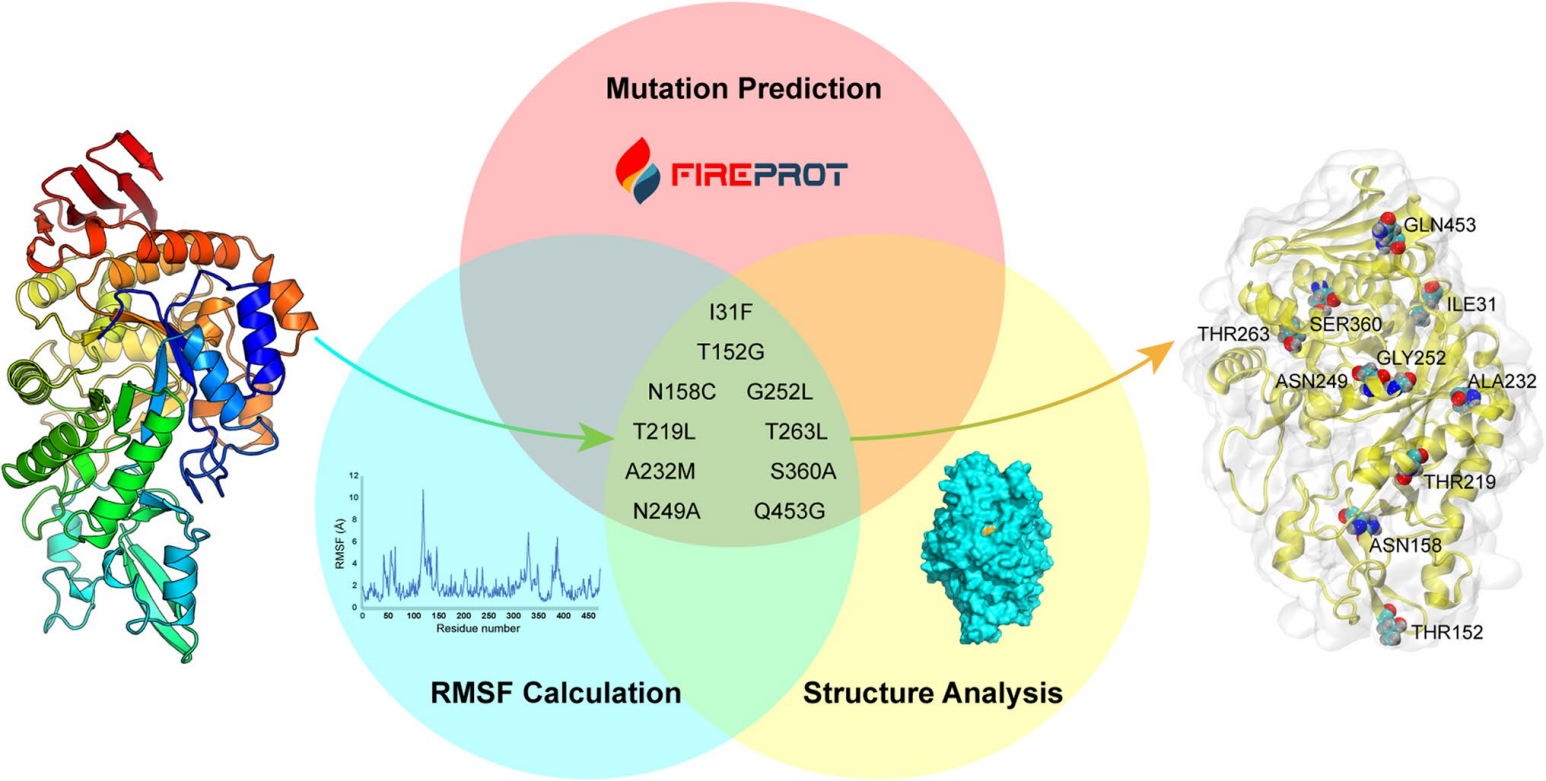
Hotspot residues prediction. The mutations were predicted by FireProt server and selected based on RMSF calculation of MD simulations and structure analysis. The 3D structure model of *Lm*SPase was shown in rainbow colors from dark blue (N-terminus) to red (C-terminus) (left). The ten hotspot residues of *Lm*SPase are shown as spheres (right)

FireProt is a web server designed to predict thermostable mutants concerning structural and evolutionary information automatedly. The FoldX (Schymkowitz et al. 2005) and Rosetta (Alford et al. 2017) tools in the server were used to compute the energy from the input structure based on authentic experimentally validated databases and simulations of the atoms’ force fields separately. FireProt also excluded strictly conserved residues that might negatively affect structural folding. Thus, FireProt was selected to generate a pool of *Lm*SPase variants that may be more stable than the WT. After calculation, a list containing 33 energy mutants and 31 evolution mutants was generated. Then, MD simulations and structural analyses of the WT were carried out. Among the mutants listed, Rosetta-based mutations with less than − 2 kcal/mol folding free energy (ΔΔG) and FoldX-based mutations with less than − 1 kcal/mol ΔΔG were selected (Bednar et al. 2015) (Table 1). In addition, after MD simulations, the mutation with a root-mean-square fluctuation (RMSF) lower than the average value of 2.12 Å at 300 K was eliminated (Fig. 1). Additionally, if the α carbon atom of the residue predicted by FireProt had a distance to the active site of *Lm*SPase less than 10 Å, this residue was removed from the mutant list. Finally, ten mutations were selected (Fig. 1).

**Table 1:** Characteristics of predicted *Lm*SPase mutants

	Enzyme	Activity before heating (%)^[a]^	Activity after heating (%)	Rosetta ΔΔG (kcal•mol^−1^)	FoldX ΔΔG (kcal•mol^−1^)
	WT	100.0 ± 1.4	33.0 ± 4.6	− ^[b]^	−
Energy-based mutants	I31F	104.0 ± 2.5	40.4 ± 1.7	− 12.03	− 3.21
	T152G	128.2 ± 7.6	N.D.^[c]^	− 41.15	− 1.31
	A232M	56.4 ± 3.8	N.D	− 37.79	− 1.54
	G252L	79.6 ± 1.5	N.D	− 2.49	− 13.48
	Q453G	147.3 ± 2.4	46.5 ± 0.2	− 13.47	− 4.34
Evolution-based mutants	N158C	69.0 ± 5.1	37.5 ± 1.6	−	− 2.77
	T219L	142.2 ± 4.1	118.8 ± 2.4	−	− 1.50
	N249A	101.9 ± 0.3	20.4 ± 0.4	−	− 1.08
	T263L	95.1 ± 9.7	51.0 ± 3.9	−	− 1.04
	S360A	108.4 ± 3.5	53.7 ± 0.8	−	− 1.44
	Mut4	97.1 ± 3.9	84.0 ± 8.2	−	−

^[a^^]^The enzyme activity is measured by determining the fructose produced, and the 1-mL reaction mixture contains 500 μL of 5% sucrose, 450 μL of 50 mM sodium phosphate buffer (pH 6.5), and 50 μL of crude extracts. The activity of crude WT before heated was taken as 100%, and other crude enzymes’ relative activity before or after heated at 50 °C for 10 min was calculated based on WT

^[b^^]^Not applicable

^[c^^]^Not detectable

### Characterization of the ten selected mutants

Ten variants were constructed and expressed in *E. coli* BL21(DE3), and the harvested bacteria were analyzed by SDS-PAGE (Figure S2). Compared with the negative control, these variants all had an obvious band at approximately 55 kDa, indicating that all the variants were expressed successfully. To determine the thermostability of the variants, we incubated the crude enzyme supernatants of WT and mutant enzymes at 50 °C for 10 min and tested the phosphorolysis of sucrose to produce α-D-G1P and fructose (Table 1). Then, the activity of the enzyme without heat treatment and the residual activity after heat treatment were determined under optimal reaction conditions. The results showed that the crude enzymes of I31F, N158C, T219L, S360A, and T263L had better thermostability than WT (Table 1). However, the activity of N158C without heat treatment decreased to 69% of that of the WT. Therefore, a combination mutation containing the four positive mutations (I31F/T219L/T263L/S360A, Mut4) was constructed and analyzed. After incubation at 50 °C for 10 min, Mut4 retained more than 50% residual activity with partial loss of its specific activity. Finally, five positive variants, I31F, T219L, S360A, T263L, and Mut4, were selected to study further.

### Characterization of the thermostable variants

**Fig. 2:**
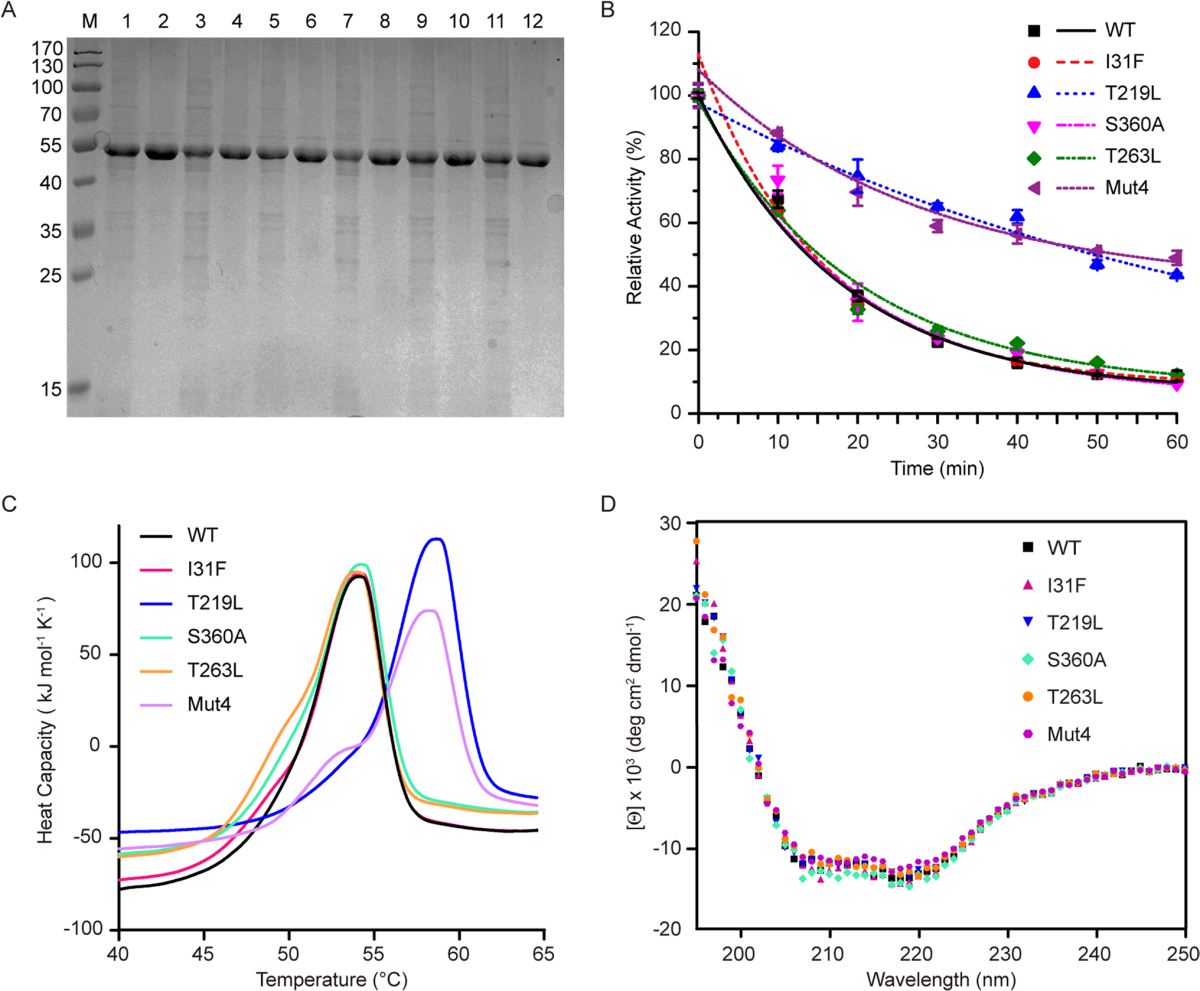
Purification and characterizations of the wild-type *Lm*SPase and variants. **A** The base lane is the cell lysate, and the even lane is the purified protein lanes: M, molecular weight marker; 1 and 2, the wild type; 3 and 4, I31F; 5 and 6, T219L; 7 and 8, S360A; 9 and 10, T263L; 11 and 12 Mut4. **B** The half-life of the WT and variants determined at 50 °C in 50 mM phosphate buffer, pH 6.5 using 0.01 mg/mL purified enzyme with the substrates sucrose and phosphates in the reaction mixture. The data points were fitted with exponential decay curves. **C** Calorimetric analysis. The *T*_m_ values of the 0.5 mg/mL purified WT and variants were determined by nano-DSC. **D** CD spectra analysis. The secondary structure of 0.2 mg/mL purified WT and variants were analyzed by CD

To further characterize the enzymes in detail, the WT and five positive variants were purified by affinity chromatography and size-exclusion chromatography, and the pure protein bands were shown in the even-numbered lanes of SDS-PAGE (Fig. 2A). To determine the thermostability of the WT *Lm*SPase and its variants, the purified enzyme solutions were adjusted to the same concentration and incubated at 50 °C, and their relative residual enzymatic activities were measured every 10 min. Compared with the half-life of WT, the variants T219L and Mut4 exhibited longer half-life (Fig. 2B). The half-life of T219L and Mut4 was calculated as 48.7 min and 53.1 min, respectively, which increased by 2.3-fold and 2.6-fold compared with that of WT, respectively (Table 2). However, the half-life of I31F, S360A, and T263L did not increase much compared to that of WT.

**Table 2:** Characteristics of purified WT and variants

Enzyme	Specific activity^[a]^ (U/mg)	*t*_1/2_ (min)	*T*_m_ (°C)
Wild-type	214.0 ± 18.7	14.6	53.4
I31F	220.3 ± 16.9	14.4	53.4
T219L	243.8 ± 23.1	48.7	58.0
S360A	230.2 ± 20.4	15.7	53.5
T263L	210.0 ± 14.2	15.1	53.0
Mut4	198.7 ± 18.8	53.1	57.4

^[a^^]^The values of purified protein represent the means ± S.D. for three independent experiments

The half-lives (*t*_1/2_) of enzymes were evaluated by incubating 0.01 mg/mL purified enzymes for 90 min in a water bath at 50 °C and testing their residual activity every 10 min. The *T*_m_ values were determined by nano-DSC, and the 0.5 mg/mL of purified enzymes were scanned from 20 to 100 °C by the scan rate at 1 °C/min

The thermodynamic stability of a protein could be evaluated by its melting temperature. The *T*_m_ values of the WT and variant enzymes were determined by nano-DSC. The *T*_m_ determinations showed that T219L and Mut4 had a stabilizing effect on *Lm*SPase (Fig. 2C). Both T219L (58.0 °C) and Mut4 (57.4 °C) had higher *T*_m_ values than that of WT (53.4 °C), while the other mutations did not seem to affect the thermodynamic stability of *Lm*SPase (Table 2). Far-UV CD spectra of the WT and variants showed that none of the mutations caused significant changes in the secondary structure (Fig. 2D).

### Catalytic production of αGG

**Fig. 3:**
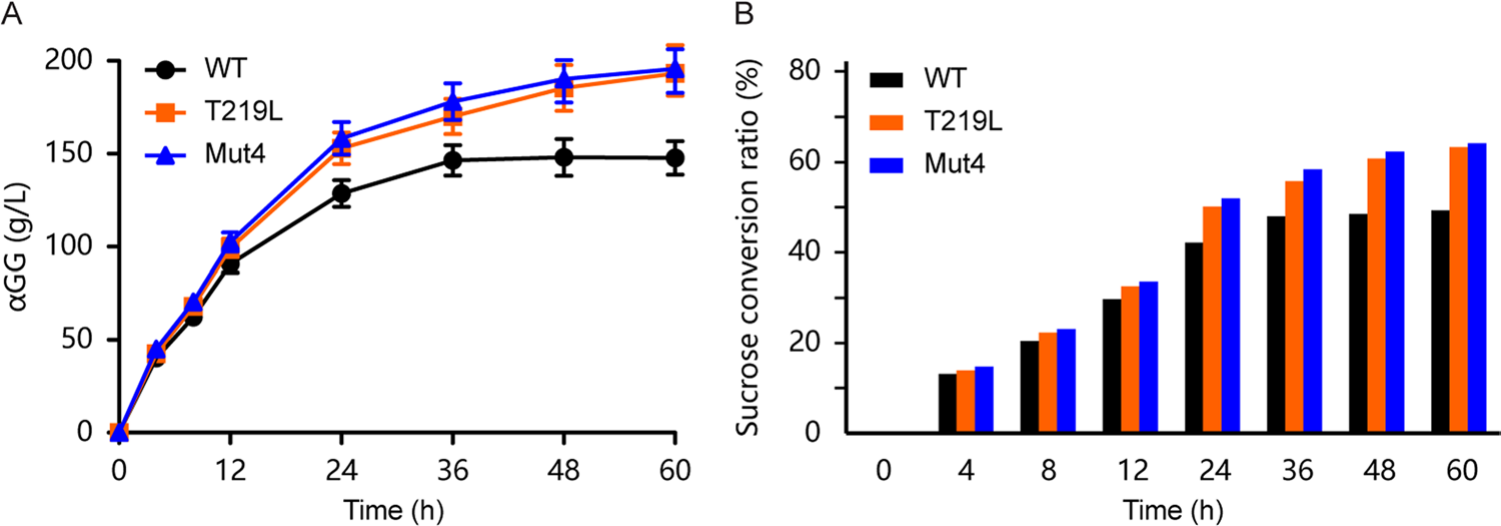
Catalytic production of αGG. **A** The αGG synthesis. The recombinant *Lm*SPases were used to catalyze sucrose and glycerol to produce αGG at 37 °C. The reaction mixture containing 1.2 mol/L sucrose, 2 mol/L glycerol, and 40 U/mL crude extracts of *Lm*SPase. **B** The molar conversion ratio of sucrose. The consumption of sucrose in reaction mixture was determined by HPLC

The αGG is an efficient moisturizing agent and has been produced in the industry. The regular reaction temperature for producing αGG is 30 °C. To investigate whether the two positive variants have advantages in the production of αGG at a higher temperature, the crude extracts of T219L, Mut4, and WT were used to synthesize αGG from 1.2 mol/L sucrose and 2 mol/L glycerol at 37 °C in MES buffer (Fig. 3A). After incubating with 40 U/mL crude extracts at 37 °C for 60 h, T219L and Mut4 achieved the product concentration of 193.2 ± 12.9 g/L and 195.8 ± 13.1 g/L, which were approximately 1.3-fold higher than that of WT (150.4 ± 10.0 g/L). During the first 24 h of the synthesis process, WT, T219L, and Mut4 catalyzed the synthesis of αGG quickly and tended to be flat at 36 h. It can be found that the αGG production of WT reached a high level at 36 h, but there was no significant increase in the following 24 h, while the two mutants still increased significantly from 36 to 60 h, indicating that the mutants with higher thermostability had advantages in the long-term catalysis process. Correspondingly, the molar conversion ratio of sucrose gradually increased with the extension of the catalytic time (Fig. 3B). After 36 h, the sucrose conversion ratio of WT slowed down, while the two mutants continued to increase. Finally, the sucrose molar conversion ratio of T219L and Mut4 reached 63.3% and 64.2%, respectively, which increased by approximately 28% compared to that of WT. As the Mut4 variant (I31F/T219L/T263L/S360A) which was constructed based on the T219L mutant showed no significant improvement on catalytic performance compared with T219L, further computational analysis was carried out to get the insight of the reason for thermostability enhancement of the T219L variant.

### RMSD analysis of thermostable variants

**Fig. 4:**
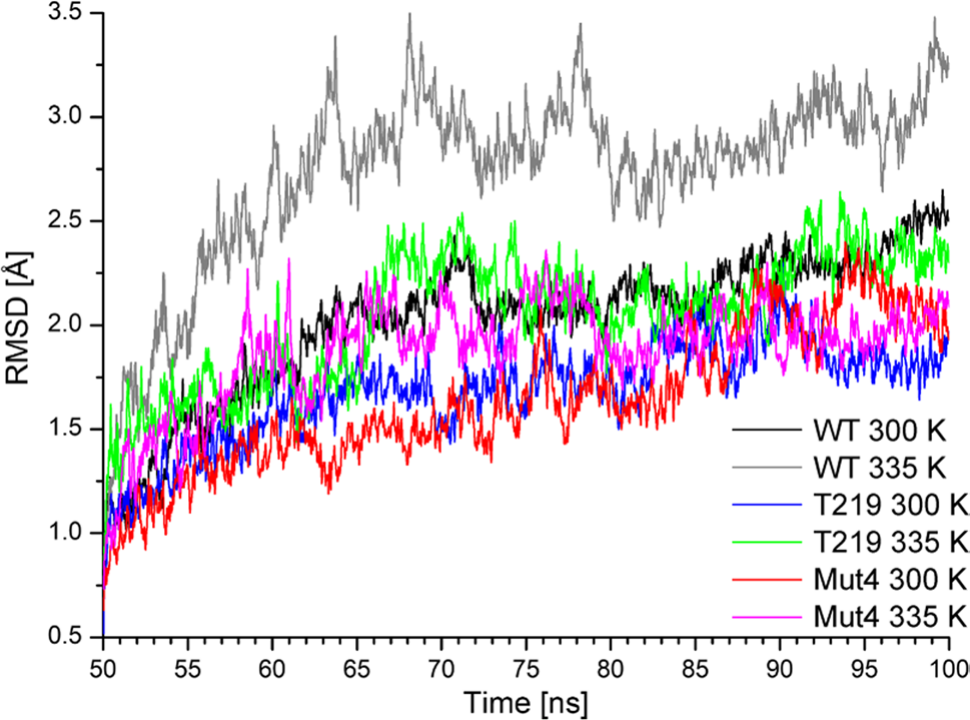
The RMSD of the WT, T219L, and Mut4. The MD simulations were performed at 300 K and 335 K for 50–100 ns. WT 300 K (black), WT 335 K (gray), T219L 300 K (blue), T219L 335 K (green), Mut4 300 K (red), Mut4 335 K (magenta). The MD simulations were calculated using NAMD 2.12 code, and the results were analyzed by VMD

In this study, MD simulations were used to analyze the structural properties of the SPase enzyme. The root-mean-square deviation (RMSD) of the enzyme backbone atoms can reflect the whole protein stability during MD simulations. The stability of WT and its mutants was analyzed by MD simulations at 300 K and 335 K. The RMSD of the WT at 300 K is between 2 and 2.5 Å, which is the typical value for globular proteins. After heating to 335 K, it can be observed that the average RMSD of the WT is distributed between 2.5 and 3.5 Å, which is higher than that at 300 K (Fig. 4). Similarly, both T219L and Mut4 have lower RMSD values at 300 K than at 335 K. Either at 300 K or at 335 K, the average RMSD values of T219L and Mut4 were lower than that of the WT, indicating that T219L and Mut4 had a more stable protein backbone.

### RMSF analysis of thermostable variants

**Fig. 5:**
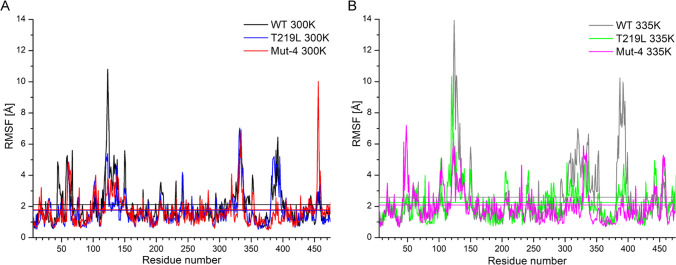
The RMSF and structure analysis. The MD simulations of WT, T219L, and Mut4 were separately performed at 300 K (**A**) and 335 K (**B**) (scales are the same). Straight horizontal lines are mean values of RMSF plots

The root-mean-square fluctuations (RMSF) reflect the possibility of movements of each amino acid in the protein, and a larger RMSF value reflects that residue has more degrees of freedom than those with smaller ones. Analysis of the RMSF plots generated based on MD simulations at 300 K and 350 K (Fig. 5A and B) showed that most parts of all variants of a protein are similar. Large changes between the WT and other variants can be observed for residues 122–125 and 455–457. Residues 128–133, 307–327, 337–357, and 382–399 had higher RMSF values of WT than those of T219L and Mut4. These regions with high RMSF values may affect the stability of SPase.

### Residues’ contact analysis of thermostable variants

Analysis of residue contacts of thermostable proteins in comparison to mesophilic proteins often shows changes in small polar amino acids to hydrophobic ones, and the increasing hydrophobicity of amino acids is one of the crucial factors in enhancing the thermostability of proteins primarily by enlarging the hydrophobic cores inside a protein, thus removing water molecules from proteins’ interior (Hait et al. 2020; Haney et al. 1999). Contact was defined as a distance lower than 5 Å between two atoms from two different amino acids. In Figure S3, contacts from three simulations at 300 K are shown for the four mutated amino acids. For the case of the T219L mutant, large changes in interactions can be observed. All residues with improved contacts with L219 are close to the active site (D196 and E237), stabilizing it by creating a hydrophobic core often present in thermophilic proteins (Hait et al. 2020), which is difficult to destroy with high temperature. In the Mut4 variant, amino acid on the position 219 has the same contacts as in a single mutation, but other residues (I31F, T263L, S360A) do not show significant changes in contacts. Thus Mut4 does not exhibit improved thermostability in comparison to the T219L variant (Figure S3).

## Discussion

SPase has a wide range of receptor specificity as it can transfer the glucose group in sucrose to different receptors. The glycosylated product has superior performance and can be used in food, pharmaceuticals, cosmetics, and other industrial fields. However, the deficient thermostability limits enzyme’s application in continuous industrial production (Xu et al. 2020). Take the production of αGG as an example. In most cases, *Lm*SPase was used to catalyze the synthesis of αGG under 30 °C (Bolivar et al. 2017; Goedl et al. 2008), and maintaining low reaction temperature by refrigeration usually causes enormous redundant energy cost. Therefore, it’s necessary to improve the thermostability of *Lm*SPase, as the αGG synthesis process usually takes several days, and enzymes are easy to gradually inactivate during such long time. Recently, improving the thermostability of SPases to enhance their usability in the industry has attracted increasing interest. For instance, Cerdobbel increased the thermostability of SPase from *Bifidobacterium adolescentis* by a combination of sequence- and structure-based mutagenesis (Cerdobbel et al. 2011). Bolivar improved the stability of SPase from *L. mesenteroides* by immobilization (Bolivar et al. 2017).

In this study, we applied a semi-rational design strategy combining computational prediction tools, structure–function analysis, and MD simulations to reduce screening scale and increased the possibility of obtaining positive mutations. The positive variant T219L with higher thermostability and activity was obtained. Although the combination mutant Mut4 also showed similarly improved performance, the analysis results showed that the critical factor is the residue 219. Additionally, the T219L variant showed advantages in the process of synthesizing αGG from sucrose and glycerol at 37 °C. At 60 h, the concentration of αGG synthesized by such mutant reached more than 190 g/L (production intensity was 3.2 g/L/h) and still gradually increased after further incubation (Fig. 3). The most recent high αGG concentration can be achieved through various protein engineering methods or immobilization of SPase (Bolivar et al. 2017). Our results provide an alternative for improving the product concentration of αGG at high temperatures compared with traditional enzyme immobilization technology.

To study the thermostability enhancement factors, MD simulations were performed. The RMSD results showed that the variant had more stable protein backbones (Fig. 4), which were in good agreement with the experimental results (Fig. 2C). The RMSF results showed that the variant had fewer flexible regions (Fig. 5), and the amino acids in these regions of WT were located on a long loop exposed to the solvent, which would cause a relatively high fluctuation. Tailoring such flexible regions might have a positive effect on the catalytic performance of SPase under harsh conditions. Similar behavior of RMSD and RMSF in the analysis of thermostable proteins is observed in studies for other enzymes like lipase (Wang et al. 2020), trehalose synthase (Chen et al. 2021), or methylenetetrahydrofolate dehydrogenase (Maiello et al. 2020). Furthermore, the intermolecular interactions between the residues were analyzed. The mutated residue of the positive variant had more interactions with the surrounding residues than that of WT, which might contribute to the enhanced thermostability of the variant. Such creation of hydrophobic cores was observed during analysis of mesophilic and thermophilic proteins sequences (Haney et al. 1999; Saelensminde et al. 2009). During the process of MD simulations, when residue is mutated into hydrophobic amino acid, the hydrophobic core can be formed, such as in neurotensin receptor (Lee et al. 2015), enzymes like trehalose synthase (Chen et al. 2021), and nitrile hydratase (Cheng et al. 2020; Guo et al. 2021). The semi-rational design approach established in the present study sheds light on the future engineering of other industrial attractive SPases, such as the one from *Bifidobacterium* *adolescentis.*

## Supplementary Information

Below is the link to the electronic supplementary material.

Supplementary file1 (PDF 575 KB)

## Data Availability

All data generated or analyzed during this study are included in this published article (and its supplementary information files).
