# Supplementary material for: Development of thermostable sucrose phosphorylase by semi-rational design for efficient biosynthesis of alpha-D-glucosylglycerol
Source: Appl Microbiol Biotechnol. 2021 Sep 20;105(19):7309–19. doi: 10.1007/s00253-021-11551-0 (PMC8494705; doi:10.1007/s00253-021-11551-0)
Supplement: Supplementary file 1 — Supplementary file1 (PDF 575 KB) [file 253_2021_11551_MOESM1_ESM.pdf]

Supplementary Materials

Applied Microbiology and Biotechnology

Development of thermostable sucrose phosphorylase by semi-rational design for efficient biosynthesis of alpha-D-Glucosylglycerol

Yuanyuan Xia[1,2], Xiaoyu Li[1,2], Linli Yang[1,2], Xiaozhou Luo[1,3], Wei Shen[1,2], Yu Cao[1,2], Lukasz Peplowski[4]*, Xianzhong Chen[1,2]*

1. Key Laboratory of Industrial Biotechnology, Ministry of Education, School of Biotechnology, Jiangnan University, 1800 Lihu Avenue, Wuxi 214122, China.

2. School of Biotechnology, Jiangnan University, Wuxi 214122, China.

3. Center for Synthetic Biochemistry, Shenzhen Institutes for Advanced Technologies, Chinese Academy of Sciences, Shenzhen 518055, China.

4. Institute of Physics, Faculty of Physics, Astronomy and Informatics, Nicolaus Copernicus University in Torun, Grudziadzka 5, 87-100 Torun, Poland.

Mailing address: School of Biotechnology, Jiangnan University, 1800 Lihu Avenue, Wuxi, Jiangsu 214122, China

Tel: +86-0510-85918122; Fax: +86-0510-85918122

* Correspondence should be addressed to Xianzhong E-mail: xzchen@jiangnan.edu.cn and Lukasz Peplowski, E-mail: drpepe@fizyka.umk.pl.

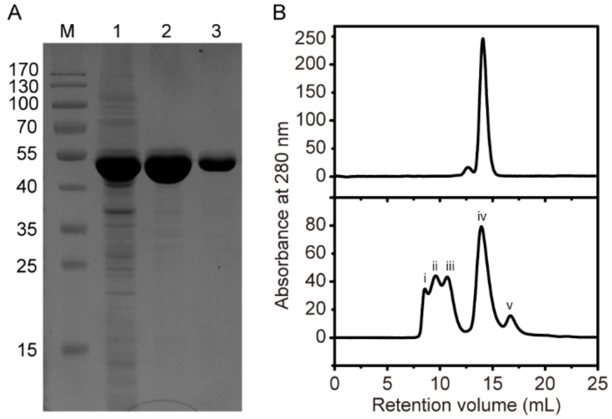

Figure S1 Expression, purification and molecular mass determination of LmSpase.

(A) SDS-PAGE analysis of recombinant *Lm*Spase. Lanes: M, protein marker; 1, the soluble cell-free extract; 2, the purified enzyme from His-tag affinity chromatography; 3, Lane 2 diluted 5 times. (B) Elution curves of *Lm*SPase (upper) and standard proteins (lower) using size-exclusion chromatography. Standard proteins: i, Thyroglobulin (669kDa, bovine thyroid); ii, Ferritin (440kDa, horse spleen); iii, Aldolase (158kDa, rabbit muscle); iv, Ovalbumin (43kDa, hen egg); v, Carbonic anhydrase (29kDa, bovine erythrocytes).

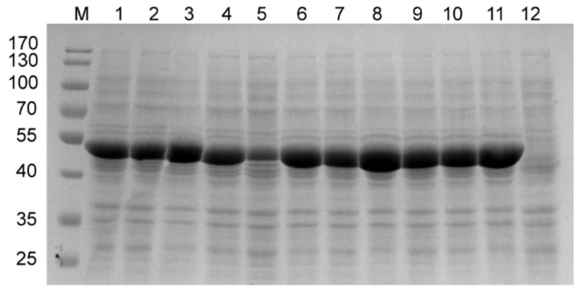

Figure S2 Protein expression of WT and its variants.

SDS-PAGE of cell lysate. Lanes: M, molecular weight marker; 1, WT; 2, I31F; 3, Q453G; 4, G252L; 5, A232M; 6, T152G; 7, N158C; 8, T219L; 9, S360A; 10, N249A; 11, T263L; 12, negative control (pET-28a plasmid).

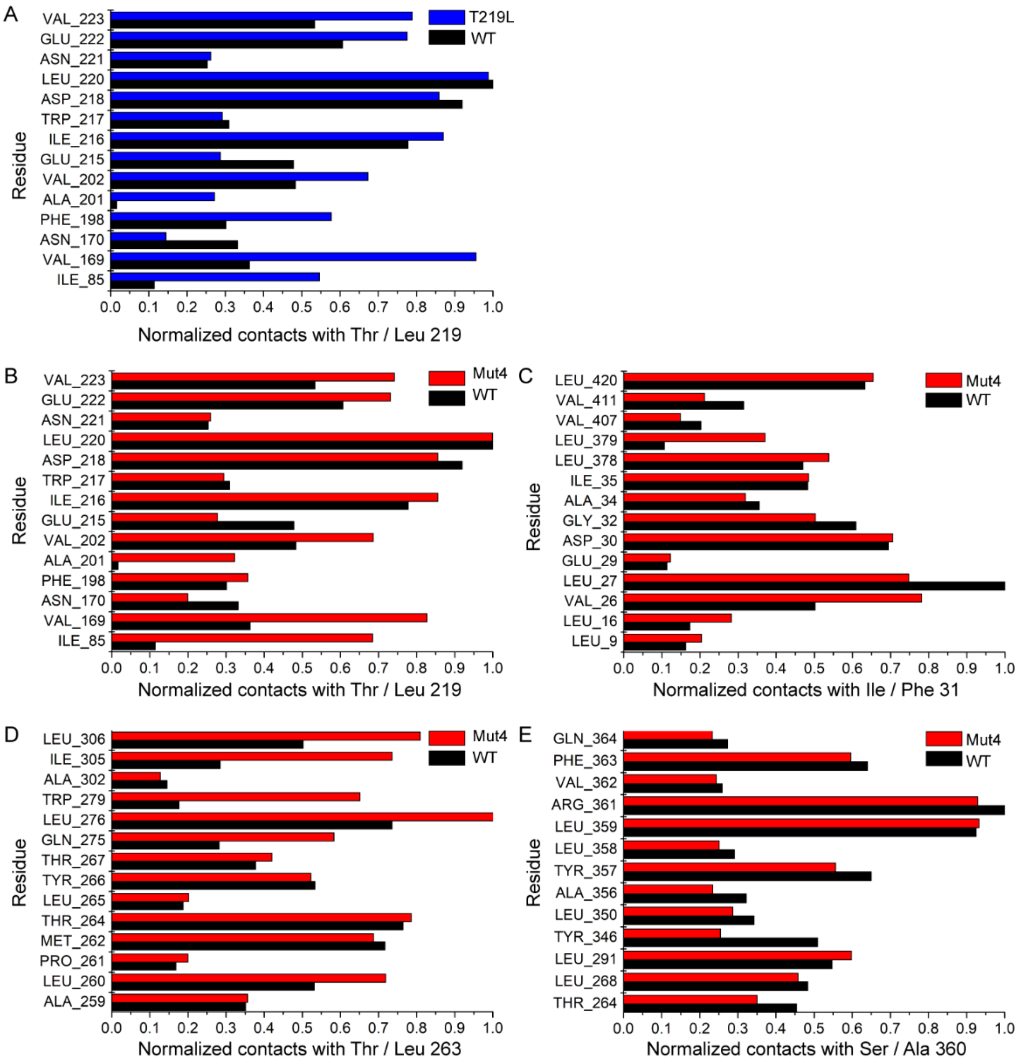

Figure S3 Normalized contacts of mutated residues calculated based on MD simulations at 300K.

(A) Contacts for residue Thr/Leu 219 of T219L(blue) and WT (black). (B) Contacts for residue Thr/Leu 219 of Mut4 (red) and WT. (C) Contacts for residue Ile/Phe 31 of Mut4 and WT. (D) Contacts for residue Thr/Leu 263 of Mut4 and WT. (E) Contacts for residue Ser/Ala 360 of Mut4 and WT.

Table S1. Oligonucleotide primers used in this study.

Primers	Sequence (5’→3’)
I31F-F	GTTCTGAAAGAAGACTTCGGTGACGCTA
I31F-R	ACCGATAGCG TCACCGAAGTCTTCTTTC
Q453G-F	ATCGTTGTTACCCGTGGCGACGAAAACG
Q453G-R	CTGACCGTTT TCGTCGCCACGGGTAACA
G252L-F	AAAATCAACGACCACCTTTACTTCACCT
G252L-R	GTCGTAGGTGAAGTAAAGGTGGTCGTTG
A232M-F	CTGACCCCGCTGAAAATGGAAATCCTGC
A232M-R	TTCCGGCAGG ATTTCCATTTTCAGCGGG
T152G-F	ACCTTCGACGACGGTGGTACCGAAAACC
T152G-R	CCACAGGTTT TCGGTACCACCGTCGTCG
N158C-F	ACCGAAAACCTGTGGTGCACCTTCGGTG
N158C-R	TTCTTCACCG AAGGTGCACC ACAGGTTT
T219L-F	CCGGAAATCTGGGACCTCCTGAACGAAG
T219L-R	ACGAACTTCG TTCAGGAGGTCCCAGATT
S360A-F	GCTGCTTACCTGCTGGCGCGTGTTTTCC
S360A-R	AACCTGGAAAACACGCGCCAGCAGGTAA
N249A-F	ATCCCGAAAAAAATCGCCGACCACGGTT
N249A-R	GAAGTAACCGTGGTCGGCGATTTTTTTC
T263L-F	TTCGCTCTGCCGATGCTCACCCTGTACA
T263L-R	CAGGGTGTACAGGGTGAGCATCGGCAGA
